# Ventilator Management of Bronchopleural Fistula Secondary to Methicillin-Resistant* Staphylococcus aureus* Necrotizing Pneumonia in a Pregnant Patient with Systemic Lupus Erythematosus

**DOI:** 10.1155/2017/1492910

**Published:** 2017-05-14

**Authors:** Ahmed F. Alohali, Saleh Abu-Daff, Kamardeen Alao, Mohammed Almaani

**Affiliations:** Department of Pulmonary and Critical Care, King Fahad Medical City, P.O. Box 59046, Riyadh 11525, Saudi Arabia

## Abstract

Managing mechanical ventilation in patient with bronchopleural fistula with coexisting acute respiratory distress syndrome is a challenging situation for the intensivist. We are reporting a case of a pregnant patient with systemic lupus erythematosus on immunosuppressive medications who developed methicillin-resistant* Staphylococcus aureus* necrotizing pneumonia complicated by bronchopleural fistula and acute respiratory distress syndrome.

## 1. Introduction

Bronchopleural fistula (BPF) is an infrequently encountered complication in intensive care units (ICU) in patients who have not undergone thoracic surgery [1] and poses difficulty in ventilator management. Coexisting acute respiratory distress syndrome (ARDS) adds to this challenge. We here report a case of a pregnant woman known to have systemic lupus erythematosus (SLE) who developed BPF as a consequence of methicillin-resistant* Staphylococcus aureus* (MRSA) necrotizing pneumonia, after which she developed ARDS.

## 2. Case Presentation

A 24-year-old 16-week pregnant woman presented at the emergency department (ED) with fever and productive cough of yellowish sputum of 3-week duration. She is a known case of SLE and was receiving prednisolone (40 mg daily), azathioprine (50 mg twice daily), and hydroxychloroquine (200 mg twice daily).

On physical examination, she was febrile with a temperature of 38.4°C. However, oxygen saturation was maintained at 96% in room air, with decreased breath sounds on the right infrascapular area.

**Figure 1:**
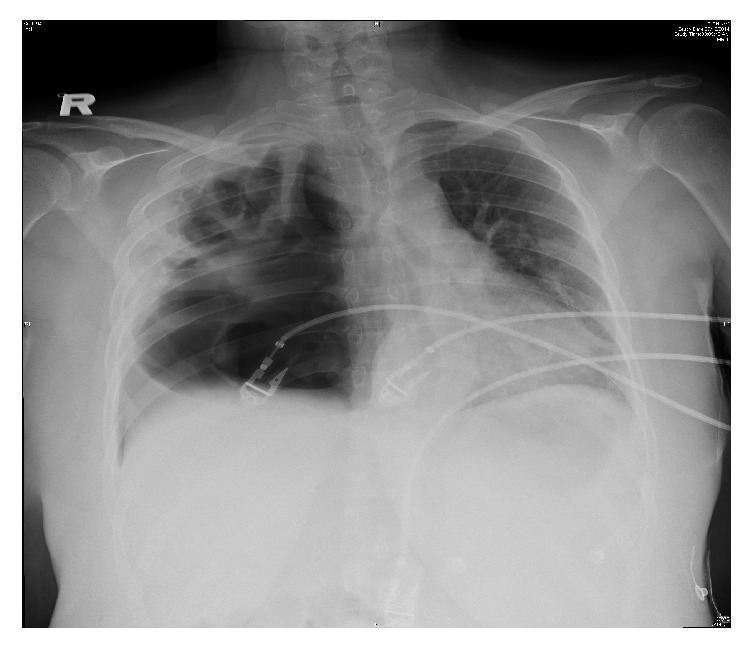
Chest X-ray showing right upper lobe cavitary lesion with a large pneumothorax and pleural effusion.

**Figure 2:**
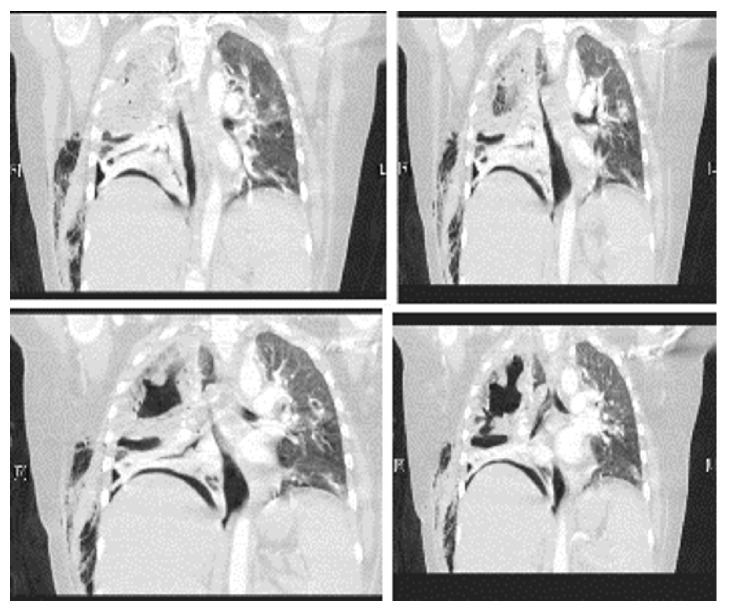
Chest computed tomography revealed multiple bilateral cavitation (more on the right), moderate right-sided pleural effusion, air-space infiltrates, and a right-sided cavitary-pleural communication.

Her chest roentgenogram on presentation (Figure 1) showed a right upper lobe cavitary lesion, with a large right pneumothorax and pleural effusion. A right-sided chest tube was inserted, which drained pus. Chest computed tomography (CT) (Figure 2) revealed multiple bilateral pulmonary cavitation (more on the right), moderate right-sided pleural effusion, air-space infiltrates, and a right-sided cavitary-pleural communication.

She was admitted to the hospital and antibiotic treatment was commenced. Her sputum and pleural fluid cultures were positive for MRSA. Subsequently, azathioprine was stopped and prednisolone was tapered off. Fetal ultrasound showed a single viable fetus with normal biometrics.

**Figure 3:**
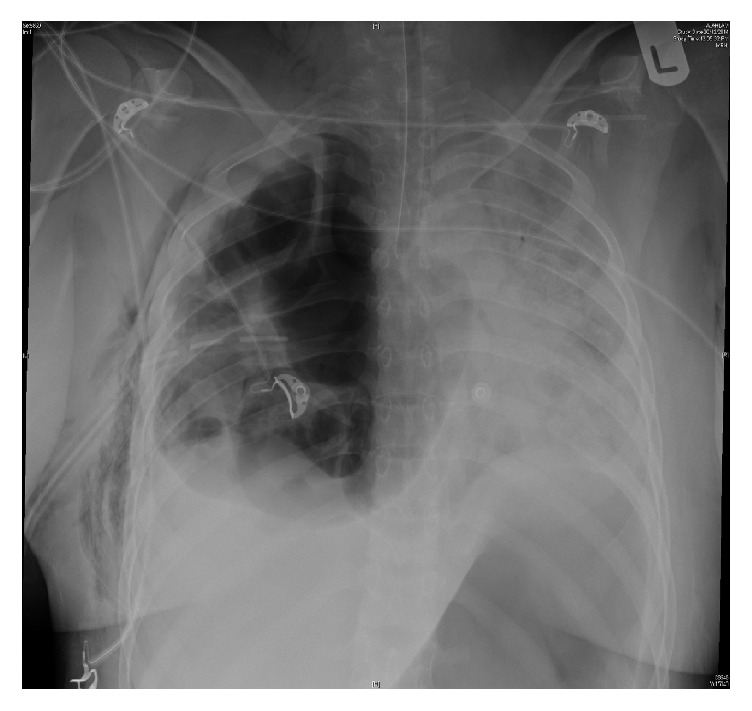
Chest X-ray showing multiple air-filled cavitary right lung lesions, right-sided pleural effusion, and progression of the air-space disease in the left lung.

During her hospital stay, she developed shortness of breath and a decrease in oxygen saturation, which required mechanical ventilation (MV) and ICU admission. Her chest roentgenogram (Figure 3) showed multiple air-filled cavitary right lung lesions, right-sided pleural effusion, and progression of the air-space disease in the left lung.

In the ICU, she was fully sedated and paralyzed, and her blood pressure was supported by means of vasopressors. Her initial mode of MV was pressure control ventilation (PCV) with inspiratory pressure of 34 cmH2O, positive end-expiratory pressure (PEEP) of 10 cmH2O, ventilator rate of 22/min, and fractional inspired oxygen concentration (FiO_2_) of 100%. After initiation of MV, the air leak through the right BPF worsened considerably. Her arterial blood gas (ABG) after initiation of MV is displayed in Table 1.

Due to sustained hypoxemia, nitric oxide (NO) was added to her MV, which was changed to high frequency oscillatory ventilation (HFOV) with a frequency of 4.5 Hz, amplitude of 70 cmH_2_O, mean air way pressure of 26 cmH_2_O, and FiO_2_ of 100%. Her gas exchange improved gradually over a few days, she was weaned off NO, and her oxygen requirements decreased. HFOV was switched to controlled mandatory ventilation (CMV) with a tidal volume (TV) of 450 mL, rate of 28/min, PEEP of 12 cmH_2_O, and FiO_2_ 45% ABG in Table 1.

Due to the persistent large air leak from the right lung, however, it was decided to initiate differential lung ventilation (DLV). The settings were as follows: left lung CMV TV 200 mL, PEEP 8 cmH2O, rate 12/min, and FiO_2_ 35%; right lung PCV pressure 15 cmH2O, PEEP 4 cmH2O, rate 12/min, and FiO_2_ 60%. The ABG obtained by these means is shown in Table 1. Air leak improved gradually over 11 days. DLV was switched to CMV with a TV of 300 mL, rate of 14/min, PEEP of 4 cmH2O, and FiO2 35%, and ABG was as summarized in Table 1.

**Figure 4:**
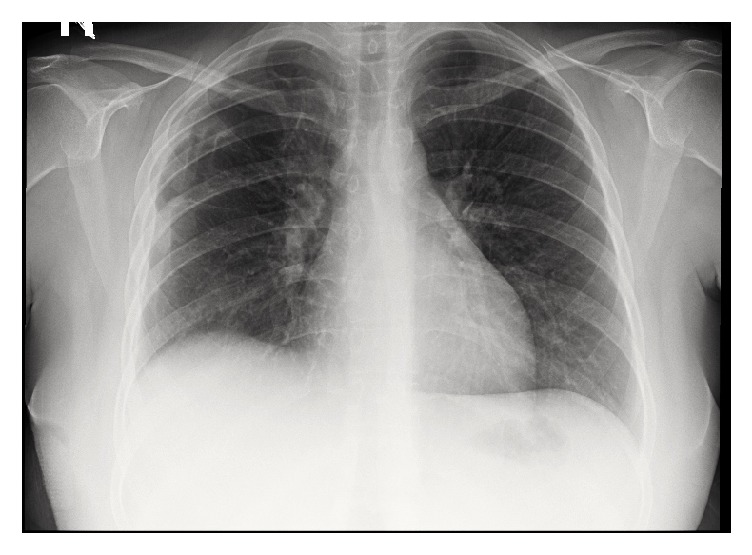
Chest X-ray on discharge showing resolution of the bilateral lung infiltrates, pleural effusions, and surgical emphysema.

**Figure 5:**
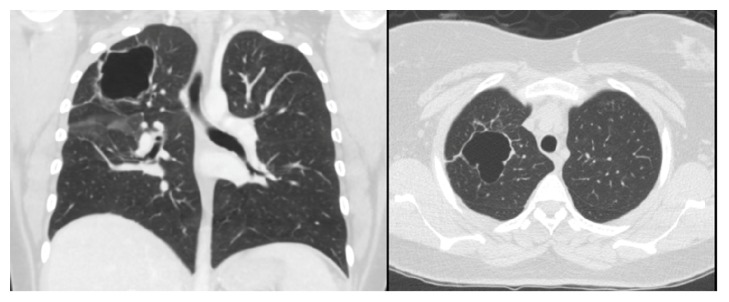
Chest CT on discharge showing resolution of the BPF and development of thin wall right upper lobe cavity.

The patient was ultimately weaned off MV and subsequently extubated. She was transferred to the ward on nasal cannula, where she gradually improved, and was later discharged home. Her chest roentgenogram and CT are shown in Figures 4 and 5.

## 3. Discussion

BPF is a direct communication between the airway and the pleural cavity. It can present acutely as a pneumothorax or subacutely or chronically as empyema [1]. Pneumonectomy and lobectomy are the most common causes of BPF, with reported incidence rates between 4.5% and 20% after pneumonectomy and 0.5% after lobectomy [2]. Other causes of BPF are listed in Table 2 [3]. The reported mortality of BPF is between 18% and 50%, with the most common causes of death being tension pneumothorax and aspiration pneumonia [4]. BPF leads to an air leak which, if significant, compromises pulmonary gas exchange, leading to respiratory acidosis and severe hypoxemia.

The management of BPF is supportive and can also involve MV and interventional management. Patients may require chest tube insertion to drain the pneumothorax and empyema. The chest tube should preferably be wide and short to minimize the flow of air through the fistula and facilitate drainage of the empyema [4] and should be used together with antibiotic management.

If the patient requires mechanical ventilation, the strategies for MV rely on minimizing the air leak and maintaining acceptable gas exchange. These can be achieved by reducing the airway pressure through the use of low TV ventilation, lowering the PEEP, shortening the inspiratory time, and reducing the respiratory rate [3]. There are no reported controlled studies comparing the various modes of conventional MV in the setting of BPF. Pierson et al. reported a case series of 39 patients with BPF maintained on conventional ventilation and showed significant mortality in patients with air leak greater than 500 mL/breath, as well as in those developing BPF late in the course of their illness.

Only two of the patients in this series developed significant respiratory acidosis that was unresponsive to conventional ventilation, despite the presence of major air leaks [5]. Litmanovitch et al. reported one case in which pressure control ventilation was successfully used in a patient with coexisting ARDS [6].

There are several reports of successful use of both high frequency jet ventilation (HFJV) and HFOV (3) in the setting of BPF. HFJV generally seems to be useful in patients with proximal BPF, without significant underlying lung disease. HFOV may be more suitable for patients with high-output BPF with poor lung compliance [4].

On the other hand, DLV facilitates optimal ventilation of the normal or less-diseased lung, while allowing maintenance of lower airway pressures on the affected side by reducing the air leak. No controlled studies and guidelines for the use of DLV in BPF are currently available. Only case reports of the use of DLV in the setting of BPF, with different modes of ventilation for each lung, are currently available in the medical literature. Cinnella et al. reported successful weaning of a case of lung contusion from DLV to conventional MV once the compliance difference became less than 20% [7].

Extracorporeal membrane oxygenation ECMO is a rapidly evolving therapy used in the management of refractory respiratory failure. There are many case report and series that reported the successful use of ECMO in cases with BPF. Daoud et al. reported successful use of arteriovenous ECMO in 5 patients via femorofemoral cannulation in the setting refractory respiratory failure associated with bronchial fistula and acute lung injury after thoracic operations and failure of conventional ventilation [8]. In a case series of 2 pediatric patients with severe* Staphylococcus aureus*-induced necrotizing, both patients were temporarily placed on venoarterial ECMO support and subsequently survived the infection [9]. In another case report, a 22-year-old female with necrotizing pneumonia and refractory hypoxemia was placed on venovenous ECMO support to minimize the risk of acute lung injury, particularly to the lesser affected lung (right). She was subsequently decannulated and had a left pneumonectomy with a favorable outcome [10]. Garlick et al. reported a successful management of 16-year-old boy posttraumatic BPF resistant to conventional ventilation with differential lung ventilation and ECMO via bicaval dual lumen catheter [11]. ECMO support can be considered for cases of complicated necrotizing pneumonia, or necrotizing pneumonia with refractory hypoxia, by itself, or with planned surgical intervention at a later date based on available equipment, medications, and personnel experience. Larger randomized studies in this regard will be helpful to further delineate the timing of ECMO, the type, the patient population in which the most favorable outcomes can be achieved. A potential limiting factor in this approach is the use of systemic anticoagulation.

In terms of definitive treatment, aggressive infection treatment and nutritional support are essential part in surgical management. Definitive surgical treatment varies according to the cause of the BPF. Most BPFs repaired surgically are in the setting of pneumonectomy, lobectomy, and wedge resection. Buttress the suture closure preferably with vascularized omental flap, muscle, or pleural flaps if the omental flaps cannot be used [12]. In critically ill patients who will not tolerate surgery especially with proximal well visualized fistula via bronchoscope, deployment of different types of sealants and valves is prescribed in the literature as case reports [1, 3].

A review of the literature revealed one report of a 32-week pregnant patient who presented with respiratory failure and septic shock secondary to a ruptured pulmonary hydatid cyst, with superimposed pulmonary tuberculosis. This patient underwent thoracotomy and cesarean section and was subsequently extubated. After procedure, she developed BPF, which was treated with chest tube insertion for 3 weeks and antimicrobials [9].

The patient presented here was unique as she had been diagnosed with SLE and was on immune suppressive medication, was pregnant, and developed BPF and ARDS, and required MV, which added to the complexity of her management. To the best of our knowledge, this is the first report of such a case.

## Figures and Tables

**Table 1:** Blood gas change on different modes of mechanical ventilation.

Mode of MV	PH	PCo2	PO2	HCO3	Sat.
PCV	7.05	85	40	23	53
HFOV	7.09	68	79	20	90
CMV 1	7.35	45	55	24	86
DLV	7.47	36	71	26	95
CMV 2	7.44	45	138	30	98

**Table 2:** 

Pulmonary resection
Persistent spontaneous pneumothorax, including ruptured bulla(e)
Necrotizing pulmonary infection
Inflammatory lung diseases
Malignancy
After chemotherapy or radiotherapy for lung cancer
Thoracic trauma
After lung transplant
ARDS
Iatrogenic (e.g., chest tube insertion and central venous line placements)
Broncholithiasis
Idiopathic
